# Sustainable Packaging Systems Using Renewable Materials

**DOI:** 10.1021/acssusresmgt.4c00453

**Published:** 2024-12-16

**Authors:** Muhammad Rabnawaz

**Affiliations:** School of Packaging, Michigan State University, East Lansing, Michigan 48824-1223, United States

**Keywords:** Plastic, Biopolymers, PLA, PHA, Lignin, Cellulose, Zero Waste

## Abstract

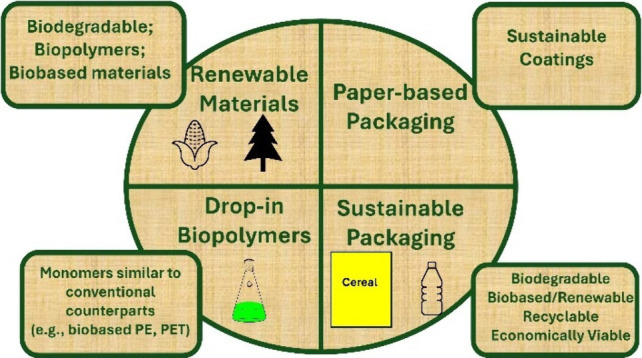

This viewpoint highlights the future of renewable materials and potential research opportunities in sustainable packaging as the world transitions from conventional petrochemical-driven packaging into more renewable-focused packaging.

## Plastic Packaging Dilemma

Packaging plays a crucial role in modern society, as it ensures storage and delivery of products such as foods and medicines, thus enhancing the safety of these products and offering consumer convenience. The global packaging market exceeded $1.01 trillion in 2021.^1^ In the U.S. alone, the packaging market reached $185 billion in 2021 and is projected to grow to $218 billion by 2027.

Plastic is the backbone of the modern packaging industry. For example, nearly one-third of all plastic produced is used in the packaging alone. This is because plastic is inexpensive, easy to process, and meets packaging requirements (e.g., desired mechanical properties, thermal sealing, tear and puncture resistance, burst resistance, liquid resistance, gas and moisture barrier properties, and so on). However, low plastic recycling rates, hindered by issues such as inconsistent compositions, contamination, presence of additives, limited collection and sorting infrastructure, and competition with low-cost virgin materials, have led to widespread landfilling and environmental accumulation of plastics.^2,3^ Unfortunately, contemporary plastics can break down into microplastics (and nanoplastics), which have now been alarmingly detected in human blood.^4^ Additionally, most conventional plastics are derived from nonrenewable petroleum resources and thus have a significant carbon footprint.

So, what’s next? It will not be possible to eliminate plastic packaging entirely, as doing so could lead to increased food spoilage and shortages in critical medical supplies. A more practical approach is to develop sustainable packaging.

## Sustainable Packaging

Sustainable packaging can be defined as packaging that does not create harmful effects before, during, or after use; is waste-free; is recyclable or biodegradable; is made from biobased or renewable materials; is safe for consumers; provides protection and shelf stability for enclosed products or goods; and is economically viable.^5^

## Renewable Materials in Sustainable Packaging

Renewable materials, like plant biomass, can be regenerated within a relatively short time frame, such as on a yearly basis. A key advantage of renewable materials is their lower carbon footprint, and some are even carbon neutral since plants absorb CO_2_ as they grow, thus balancing their overall environmental impact. Additionally, many renewable materials are biodegradable, meaning they do not produce persistent microplastics and are safer for consumers and the environment. Overall, renewable materials usage in packaging can help to decarbonise and defossilized the packaging sector.

Historically, renewable polymers were the leading plastics for packaging. For example, cellulose acetate, commonly known as cellophane, was used to create the first transparent packaging films.^6^ However, with the discovery of synthetic plastics offering lower costs, rapid scalability, and tailorable performance, the use of renewable plastics in packaging declined from the 1960s through the 1980s in favor of petrochemical-based plastics. Recognizing the environmental challenges posed by petrochemical plastics, interest in renewable materials as packaging options has surged many times over the past three decades. The urgency of this issue has grown recently due to the potentially grave implications of climate change and microplastics.

Renewable materials used in packaging are generally categorized into two types, including biopolymers and biobased materials. The first category includes various biopolymers, such as cellulose, zein, starch, lignin, and polyhydroxyalkanoates (PHAs). These naturally derived polymers are used both with and without modification. These materials are biodegradable, and thus, they do not form persistent microplastics. Despite their environmental and health benefits, biopolymers offer poor performance and processability. For example, cellophanes are often coated with polyvinylidene chloride (PVDC) to obtain desirable water resistance, thermal sealing, and oxygen barrier properties.

However, not all biopolymers are difficult to melt-process or lack performance. For example, PHAs can be melt-processed similarly to conventional polymers and thus can be transformed into different packaging articles. However, PHAs are relatively expensive, have limited availability, and offer only modest gas and moisture barrier properties, which makes them less competitive with petrochemical-based plastics, such as polyethylene (PE), polypropylene (PP), and poly(ethylene terephthalate) (PET). Continued research is needed to enhance the performance, processability, and cost-effectiveness of biopolymers and to facilitate their modification.

Biobased polymers such as polylactic acid (PLA) are synthesized from naturally derived feedstock chemicals. PLA offers numerous advantages, including good melt processability (comparable with PE and PP), industrially compostable, and cost parity with the PP, making it a leading contender in sustainable packaging. However, PLA has its challenges. When it leaks into the environment, it can form persistent microplastics (which remain in the environment for a long time) because it only slowly biodegrades in ocean and soil environments.^7^ Unfortunately, PLA lacks high gas and moisture barrier properties, and thus, it is used primarily for cups and plates either as solo material or after being coated onto paper.

## Drop-In Biobased Polymers

“Drop-in” refers to the synthesis of conventional petrochemical-derived plastics, such as PET, high-density polyethylene (HDPE), etc., using renewable monomers. The monomers themselves are otherwise identical to their conventional counterparts, but they are derived from natural materials rather than petrochemicals. For example, biobased versions of PE, PET, and PP are available, though currently at a higher cost. As is the case with their petrochemical counterparts, biobased PE, PET, and PP are nonbiodegradable. Also, the environmental benefits, particularly the carbon footprint of drop-in polymers, vary based on the difficulty of sourcing and producing biobased monomers for drop-in polymers. For instance, biobased PET can have a higher CO_2_ footprint than petrochemical-derived PET.^8^ On the contrary, biobased PE can have a lower (even negative) CO_2_ footprint compared to its petrochemical counterpart.^8^

## Paper as a Sustainable Substrate

Over the last few years, there has been a shift toward paper-based materials as alternatives for single-use plastic packaging. Paper is renewable, biodegradable, and cost-effective, but it cannot replace plastics because it is a porous fiber network. A coating layer is needed in order to impart paper with sealing properties and resistance against liquids. Consequently, coated and laminated papers are widely used in packaging for various packaging applications. However, these coatings must be separated from the paper pulp during recycling, which adds costs to this process.

Biopolymers like zein, starch, chitosan, and modified plant oils are promising alternatives for paper coatings that can render paper recyclable, biodegradable, and environmentally safe.^9−12^ One particularly promising option is polymerizable plant-based oils because they provide excellent water and oil resistance.^13^ Recent advances have led to waterborne paper coating formulations.^14^ However, cross-linked coatings have poor thermal sealing properties, and coated paper is difficult to recycle. Thus, further research is needed to address these barriers.

## Moving Forward

Sustainable packaging design is built on three foundational pillars, prioritized in the following order: environmental sustainability > performance > and cost-effectiveness.^5^ Renewable materials generally have a lower carbon footprint, as they absorb CO_2_ during production. However, they must degrade safely to avoid creating microplastics—a challenge for some renewable polymers. Renewable materials such as PHAs, plant oils, zein, starch, and cellulose are promising candidates for sustainable packaging development and application. Chitosan is not a promising packaging material due to its limited availability. Further research is also needed to develop cost-effective chemical modifications for natural polymers to make renewable materials to compete with petrochemical-derived plastics. It is also recommended that a step-by-step or phased strategy be adopted. Addressing these challenges through in-depth and commercially relevant approaches will catalyze the widespread adoption of renewable materials in packaging, a market sector that has surpassed $1.01 trillion in 2021.^1^ Priority should be given to addressing significant and easily solvable problems in renewable packaging to enable the rapid entry of renewable materials into the market. At the same time, more complex and challenging issues should be approached with a mid-to-long-term perspective.
